# Comparison of time-matched aerobic, resistance or combined exercise training in women living with obesity: a protocol for a pilot randomised controlled trial—the EXOFFIT (Exercise for Obesity in Females to increase Fitness) study

**DOI:** 10.1186/s40814-022-01003-5

**Published:** 2022-02-21

**Authors:** Mary E. Davis, Catherine Blake, Caitriona Cunningham, Brian P. Carson, Gráinne O’Donoghue

**Affiliations:** 1 Department: School of Public Health, Physiotherapy and Sports Science, University College Dublin, Dublin 4, Ireland D04 V1W8; 2 Department of Physical Education and Sport Sciences, University of Limerick, Limerick, Ireland

**Keywords:** Obesity, Women, Exercise, Fitness, Training

## Abstract

**Introduction:**

Obesity in women has more than doubled in the past thirty years. Increasing research suggests that increased cardiorespiratory fitness (CRF) can largely attenuate the negative health risks associated with obesity. Though previous literature suggests that combined training may be the most effective for improving CRF in adults with obesity, there is minimal research investigating the efficacy of combined and resistance programmes in women with obesity. This article outlines a protocol for a parallel pilot study which aims to evaluate the feasibility and efficacy of three exercise modalities in women with obesity for increasing CRF and strength and improving body composition and other health outcomes (i.e. quality of life).

**Methods and analysis:**

Sixty women (aged 18–50) with obesity (body mass index [BMI] ≥ 30 and/or waist circumference ≥ 88 cm) who are physically inactive, have no unstable health conditions and are safe to exercise will be recruited from September 2021 to December 2022. The main outcome will be feasibility and acceptability of the intervention and procedures. Trial feasibility outcomes will be evaluated to determine if a definitive trial should be undertaken. Trial acceptability will be explored through follow-up qualitative interviews with participants. Secondary outcomes will include CRF (predicted VO_2_ max), anthropometrics (i.e. BMI), strength (5RM bench press, leg dynamometry, grip strength) and other health outcomes (i.e., pain). Participants will be block randomised into one of four trial arms (aerobic exercise, resistance training and combined training groups, non-active control group) and measurements will be completed pre- and post-intervention. The exercise groups will receive an individualised supervised exercise programme for 3× sessions/week for 12 weeks. The change in mean values before and after intervention will be calculated for primary and secondary outcomes. ANOVA and *t*-tests will be applied to evaluate within-group and between-group differences. If sufficient participants are recruited, the data will be analysed using ANCOVA with the age and BMI as covariates.

**Discussion:**

This pilot will provide data on the feasibility and acceptability of trial procedures and of the programmes’ three progressive time-matched exercise interventions (aerobic, resistance and combined) for women living with obesity, which will help inform future research and the potential development of a full-scale randomised clinical trial.

**Trial registration:**

ISRCTN, ISRCTN13517067. Registered 16 November 2021—retrospectively registered.

## Background

Despite decades of awareness of the obesity challenge, this chronic disease continues to prevail as one of the world’s leading causes of morbidity and mortality [1, 2]. Women, in particular, appear more susceptible to obesity, with the prevalence of female obesity having more than doubled in the past 30 years and the prevalence of morbid obesity in women more than twice that recorded in men [3]. Evidence suggests that among adults, young women of childbearing age (18–44 years) are the most at risk of developing obesity [4, 5], with this cohort demonstrating the highest rate of weight gain [6–10]. Furthermore, the adverse effects associated with obesity appear to be greater in women, with the risks of developing cancer and cardiovascular and metabolic disorders significantly higher than observed in men [11–15]. Mounting evidence highlights the strong association between excessive weight gained during early childbearing years and longer-term adverse health outcomes [4, 16–18].

Encouragingly, increasing literature indicates that improvements in cardiorespiratory fitness (CRF), attainable through exercise, can largely attenuate the health-related risk factors, regardless of obesity severity [19–23]. Noteworthy, however, is that women, particularly young women, are significantly less active than their male counterparts [19, 20, 24, 25]. Globally an estimated 1 in 3 women do not meet generic exercise guidelines (150 min/week of moderate-intensity aerobic exercise, × 2 days/week of moderate-high intensity resistance training), with some country-specific statistics indicating that closer to 1 in 2 women aged 18–54 not meeting these recommendations [26–30]. This physical inactivity has been shown to strongly correlate with excessive weight gain in the short term and the development of cardiovascular risk factors over the longer term in this cohort [5, 31–33]. Alongside physiological differences between men and women (i.e. body size, muscle and fat mass, cardiac output etc.) [33–36], physical inactivity as a behaviour, has been identified as a stronger determinant of CRF in women [37], with the literature reporting an estimated average difference in CRF up to 20% between the sexes (in both active and inactive adults) [37–39].

To date, it is unclear how this difference in CRF translates to sex-specific responses to exercise training in the context of obesity. Evidence based on normal weight, healthy adults, suggests that males demonstrate greater increases in both absolute and relative maximal oxygen uptake (VO_2_ max) in response to training, and highlights a more blunted adaption to training in females [40, 41]. Most literature investigating the efficacy of exercise in women with obesity has primarily focused upon the prescription of interventions based upon generic physical activity guidelines, where moderate-intensity aerobic exercise is advocated. Although most of the studies focus on weight loss, the few that include CRF as an outcome measure, have observed small to moderate improvements in CRF [42–45]. Previous literature, including a recent network meta-analysis, indicate that combined (aerobic and resistance) interventions (particularly high intensity) are the most promising for improving CRF and body composition in adults living with obesity [46, 47]. However, most of the available studies which investigated resistance training or combined training were in men. Additionally, a recent systematic review and meta-analysis (conducted by this protocol’s author [M.E.D] and colleagues) which focused solely on women with obesity, further highlighted both the paucity of well-designed exercise intervention studies focused on young women with obesity and the lack of research investigating CRF and other measures of health such as quality of life, pain and mood.

### Aims and objectives

Therefore, the primary aim is to evaluate the feasibility and acceptability of prescribed time-matched aerobic, resistance and combined interventions (targeting changes in cardiorespiratory fitness) in women with obesity and to inform whether a future definitive trial could or should be undertaken. We will assess recruitment challenges; retention rates, attendance rates and adherence of participants to prescribed programme, incidence of adverse events and participant’s experience of the intervention and the acceptability of trial procedures and the programmes. We will also collect data from which a power calculation can be based to determine the number of participants needed per group in order to achieve significance in a potential follow-up randomised controlled trial (RCT).

The secondary objectives are to determine the mean difference between groups (three exercise groups and a non-exercise control group) at 12 weeks in:

(i) CRF (predicted VO_2_ max)
(ii) anthropometric outcomes (Body mass index (BMI), percentage body fat (%BF), lean mass (LM), fat mass (FM), waist-hip ratio (WHR) and waist circumference (WC)
(iii) strength (five repetition maximum [5RM] bench press, leg dynamometry, grip strength)
(iv) self-reported quality of life, physical activity, sedentary time, sleep, mood and pain

### Hypotheses tested

Primary null hypothesis: Post intervention, there is no difference in outcomes (CRF, strength, body composition, self-reported) between participants randomised to the exercise interventions and the control.

Secondary null hypothesis: Post intervention, there is no difference in outcomes (CRF, strength, body composition) between participants randomised to the aerobic exercise, resistance training and combined training groups.

## Methods

### Quantitative study

#### Trial design

**Fig. 1:**
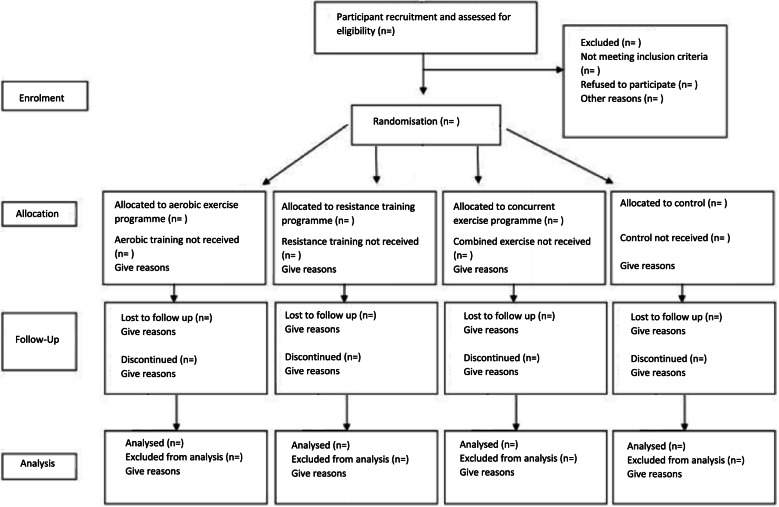
Participant flow through the pilot (based on CONSORT statement)

**Fig. 2:**
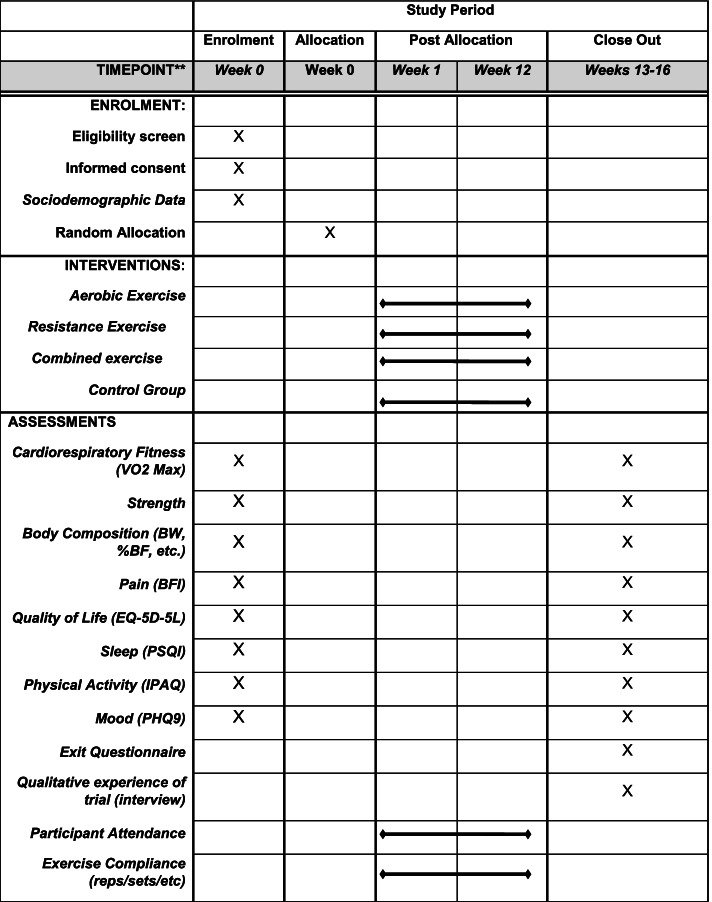
Schedule of enrolment, interventions, and assessments (based on SPIRIT protocol)

This four-arm parallel pilot study has been approved by University College Dublin (UCD) Human Research Ethics Committee (LS-21-59-Davis-ODonoghue) and will be reported in line with the Consolidated Standards of Reporting Trials (CONSORT) guidelines extension for randomised pilot and feasibility trials [48] and the Standard Protocol Items: Recommendations for Interventional Trials (SPIRIT) [49]. Following initial screening, the participants will be randomised with an allocation ratio of 1:1:1:1 into one of the three time-matched exercise modes or control (Figs. 1 and 2).

1. Aerobic: progress to 150 min/week of continuous aerobic exercise at 75–80% HRR
2. Resistance training: progress to 150 min/week of 3–6 × 12 reps of resistance exercise at 75–80% 1-RM
3. Combined training: progress to a 50:50 split of combined aerobic (75–80% HRR) and resistance (3–6 × 12 reps, 3 exercises at 75–80% 1RM) training for 150 min/week
4. Control: non-exercise group (participants to maintain baseline physical activity levels)

#### Study setting

The study will be conducted in University College Dublin (UCD) in Dublin, Ireland from September 2021 to December 2022. Baseline/follow-up assessments and all exercise intervention arms will take place in UCD Institute for Sport and Health (ISH).

#### Participants

Participants will be recruited using a flyer which will be distributed in UCD internal networks, on social media platforms and in local areas (clinics, health centres, pharmacies) and shared with the chairperson of the Irish Coalition for People Living with Obesity (ICPO) with its members. Interested participants will be encouraged to contact the trial coordinator (M.E.D) who will complete eligibility screening over the telephone using the criteria detailed in Table 1 to evaluate a participant’s eligibility and individual safety for starting an exercise programme using the Physical Activity Readiness Questionnaire for Everyone (PAR-Q+). If screened as eligible, potential participants will receive the study information sheet and consent form by email or by post (preferred method). All consenting participants will be invited to ISH for baseline assessment a minimum of seven days later (cooling off period) (Fig. 2).

**Table 1:** Eligibility criteria for inclusion in trial

Criterion	Characteristics of eligible participants
1	Female aged 18-50 years at time of consent
2	Have a Body Mass Index (BMI) ≥ 30 kg m^−2^ and /or a waist circumference > 88 cm
3	Are currently physically inactive (exercising less than 150 min/week)
4	Have not undergone weight loss surgery or another surgery in the past 3 months
5	Not pregnant (or within 6 months post-pregnancy) or lactating
6	Do not have a significant mental illness or cognitive deficits
7	Are not participating in another trial (exercise-based or targeting weight-loss) at time of consent
8	Are not contraindicated or no clinician (i.e. GP) has advised them against exercising (i.e. chest pain during activity or at rest, severe hypertension etc.)
9	Do not have an unstable cardiovascular, respiratory, renal or hepatic condition

#### Baseline assessment

On arrival at UCD ISH, the trial coordinator will meet potential participants and provide an overview of the study, an introduction to the tests that will be performed, the equipment that will be used and the purpose of each test. Participants will have the opportunity to ask any questions about the study at this time. Signed consent forms will be collected from willing participants. No study procedures will commence until written consent is obtained.

Sociodemographic and related health information of participants will be collected, including education employment status, medical and exercise participation history. Participants resting blood pressure and heart rate will be measured three times and an average calculated. Blood pressure will be measured on the upper arm using a wireless blood pressure monitor (make: Omron; model: Evolv) prior to the test. For participants where the bicep cuff is undersized, a wrist monitor will be used (make: Omron; model: R57 Intelli IT). In order to be eligible to continue to exercise testing, participants must have a resting blood pressure ≤ 160/100 (grade 2 hypertension) [50]. Once there are no contraindications to exercise testing recorded [51], objective measures of anthropometry, strength and CRF will follow.

##### Anthropometry

The following anthropometric/body composition variables will be collected (body mass index [BMI], body weight [BW], body fat percentage [%BF], waist circumference [WC], lean mass [LM], fat-free mass [FFM], waist-hip ratio [WHR], height). All outcomes will be measured using standard procedure with participants dressed in light, close-fitted clothes and with bare feet. Height, WC and BW will be measured to the nearest 0.1cm and 0.1kg respectively using a calibrated scales, stadiometer and tape measure respectively. Waist circumference will be recorded midway between the top of the pelvic bone and the bottom margin of the last rib using a tape measure [52]. Whole body composition will be measured using a bioelectrical impedance body composition analyser (seca mBCA 515).

##### Muscular strength

In order to measure strength, a 5RM bench press, leg dynamometry and grip strength will be used. To measure repetition maximum for bench press, participants will be instructed on proper form for this exercise and guided through progressive lifts where the weight will be increased in increments of 1.25–2.5 kg as needed until the participant reaches a weight at which they cannot perform all 5 reps of the movement maintaining good form. Fifty seconds of rest will be provided between attempts.

Leg strength will be measured using a back-leg strength dynamometer (Takei Back-D TKK 5402, Takei Scientific Instruments, Japan). Participants will be instructed to stand on the base of the machine, with feet shoulder width apart and their back bent slightly forward at the hips. The length of the chain will be adjusted so that the base of the bar rests superior to the anterior pole of the patella. Looking forward with a straight back, participants will be instructed to pull on the chain as hard as they can for 3 s in one fluid movement. Participants will be instructed to perform this test three times, with a rest of 50 s after each attempt. The maximum weight (kg) of all three attempts will be recorded. Grip strength will be measured using a hand-held dynamometer (Jamar Hydraulic Hand Dynamometer, Performance Health Supply, USA). To perform this measure, participants will be placed in sitting and the dominant arm supported on a side table and positioned with shoulder adducted, neutrally rotated, elbow flexed at 90°, forearm in neutral position and wrist between 0–30° dorsiflexion and 0–15° ulnar deviation [53]. The dynamometer handle will be set to the second handle position from the inside. Participants will be instructed to squeeze the handle for 3 s and will perform the test three times, with a rest of 50 s between attempts. The maximum weight (kg) of all three attempts will be recorded.

##### Cardiorespiratory fitness

Given this study will recruit some high-risk participants (obesity class I: BMI 30–30.9 kg/m^2^, obesity class II: BMI 35–39.9 kg/m^2^ and obesity class III: BMI ≥ 40kg/m^2^) [51, 54] and in order to protect participant safety, a submaximal graded walking treadmill (make: h/p/cosmos; model: quasar) test will be conducted and used to estimate VO_2_ max. Guided by the Balke-Ware protocol for females [55], participants will warm-up for 4 min starting at a speed of 3.2km/hr and an incline of 0% and progressing to 3.8km/h by the end of the warmup, in line with the speed recommended for adults with obesity in the modified Balke protocol [56] With the speed remaining consistent, the gradient will then be increased by 2.5% every 3 min up until a gradient of 25%. If the participant has not achieved any of the criteria for test termination at this stage, the speed of the treadmill will then be increased by 0.3km/h every 3 min until they do so. The test will be deemed complete and terminated once the participant reaches 85% of their age-predicted HR_max_. Alternatively, the test will be terminated before the participant reaches 85% of age-predicted HR_max_ if (i) they report a rating of perceived exertion (RPE) ≥ 18 or (ii) reach volitional exhaustion. An RPE cut-off was included as a potential reason for test termination given that RPE ≥ 17–19 has both been utilised as a criterion for achieving VO_2_ max [57] and previously as a criterion for the termination of a submaximal Balke treadmill protocol in a clinical population [58, 59]. Furthermore, research indicates that RPE > 17 reasonably correlates with VO_2_ max and can be used to predict VO_2_ max with acceptable accuracy [60].

VO_2_ max will be estimated using the Fitness Registry and the Importance of Exercise National Database (FRIEND) equation (VO_2_ max = speed × (0.17 + fractional grade × 0.79) + 3.5) [61]. Gas exchange including O2 and CO2 concentrations, and respiratory exchange ratio (RER) will be measured using a gas analysis system (make: COSMED; make: Quark CPET, Rome, Italy). Heart rate will be measured every minute throughout the test by a heart rate monitor (make: Polar; model: Wind with WearLink chest belt). As CRF is being measured using a sub-maximal protocol, electrocardiograms will not be used during testing. Rating of perceived exertion (RPE) will be measured using the Borg RPE scale every minute of the test [62].

##### Subjective self-report questionnaires

All participants will be issued a combined set of valid and reliable self-reported outcome questionnaires detailed in Table 2. These will take approximately 30 min to complete. The participant will be given the option to complete these surveys online or in paper format. Participants who wish to complete the questionnaires online will be sent an email with the link and the option to complete them either in advance or following their appointment in ISH. The self-reported measures are in line with the core outcome set for weight management interventions in adults with obesity [63, 64]. In addition, pain was included as an outcome given the strong association between chronic pain and obesity and the potential impact of pain on exercise adherence [65, 66].

**Table 2:** Primary and secondary outcome measures

Outcomes	Measure(s)
Anthropometrics	BMI, body weight, % body fat, waist circumference, fat-free mass, lean mass, waist hip ratio, height
Cardiorespiratory Fitness	Predicted VO_2_ max
Muscle strength	5RM bench press, leg dynamometry, grip strength
Pain	Brief Pain Inventory (Short form)
Health-related quality of life	EuroQol-5D-5L (EQ-5D-5L) questionnaire
Mood	Patient Health Questionnaire-9 (PHQ-9)
Self-reported physical activity & sedentary time	International Physical Activity Questionnaire (IPAQ) (Short Form)
Sleep	Pittsburgh Sleep Quality Index

#### Randomisation

A member of the research team, with no involvement in the baseline/follow-up assessments or delivery of the interventions will independently assign all consenting participants a unique number using a random number table. Another independent investigator will utilise a computer-generated random allocation sequence to allocate numbered participants to each of the trial arms. Participants will be block randomised in blocks of four. The trial coordinator (M.E.D) will then contact this independent investigator to obtain each participant’s random group allocation.

#### Interventions

All exercise interventions are 12-week programmes, comprised of 3 × 50-min sessions per week. All sessions will be supervised by trained exercise monitors/instructors (monitor: participant ratio 1:1) and will start and end with warm-up and cool-down exercises (i.e. low-intensity cycling or cross trainer, general whole body movements and stretches).

##### Aerobic

This intervention consists of 3 × 50-min sessions per week of progressive continuous aerobic exercise, completed on a cross-trainer or stationary bike or combination of both. Over the course of weeks 1 to 6, session length will gradually increase (in 5-min blocks) until participants can perform 3 × 50-min sessions in week 7. The programme will progressively increase in intensity from weeks 1 to 12 (Table 3), starting at an intensity of 40–50% HRR (weeks 1–2) and finishing at an intensity of 75–80% HRR (weeks 11–12). Participants will wear heart rate monitors and use the RPE scale to monitor intensity levels (make: Garmin; model: Forerunner 45).

**Table 3:** Progression plan from week 1 to week 12 for all exercise groups

Summary description of exercise interventions [frequency, intensity, time and type (F.I.T.T.)]						
Trial arm	Weeks 1–2	Weeks 3–4	Weeks 5–6	Weeks 7–8	Weeks 9–10	Weeks 11–12
Aerobic	3 sessions/week Low Intensity (40–50% HRR)^a^ Continuous aerobic exercise 20–30 min^b^	3 sessions/week Low–moderate intensity (50–55% HRR)^a^ Continuous aerobic exercise 30–40 min^b^	3 sessions/week Low–moderate Intensity (55–60% HRR)^a^ Continuous aerobic exercise 40–50 min^b^	3 sessions/week Moderate intensity (60–70% HRR)^a^ Continuous aerobic exercise 50 min	3 sessions/week Moderate–vigorous intensity (70–75% HRR)^a^ Continuous aerobic exercise 50 min	3 sessions/week Vigorous intensity (75–80% HRR)^a^ Continuous aerobic exercise 50 min
Resistance	3 sessions/week Low load (40–50% 1-RM)^a^ Resistance training 20–30 min^b^ 2 × 12 reps (6 exercises)	3 sessions/week Low–moderate load (50–55% 1-RM)^a^ Resistance training 30–40 min^b^ 3 × 12 reps (6 exercises)	3 sessions/week Low-moderate load (55–60% 1-RM)^a^ Resistance training 40–50 min^b^ 3–6 × 12 reps (6 exercises)	3 sessions/week Moderate load (60–65% 1-RM)^a^ Resistance training 50 min; 3–6 × 12 reps (6 exercises)	3 sessions/week Moderate–high load (70–75% 1-RM)^a^ Resistance training 50 min; 3–6 × 12 reps (6 exercises)	3 sessions/week High load (75–80% 1-RM)^a^ Resistance training 50 min; 3–6 × 12 reps (6 exercises)
Combined	3 sessions/week **Aerobic component** Low intensity (40–50% HRR)^a^ Continuous aerobic exercise 10–15 min **Resistance component** Low load (40–50% 1-RM)^a^ Resistance training 10–15 min; 2 × 12 reps (3 exercises)	3 sessions/week **Aerobic component** Low–moderate intensity (50–55% HRR)^a^ Continuous aerobic exercise 15–20 min **Resistance component** Low–moderate load (50–55% 1-RM)^a^ Resistance training 15–20 min; 3 × 12 reps (3 exercises)	3 sessions/week **Aerobic component** Low–moderate Intensity (55–60% HRR)^a^ Continuous aerobic exercise 20–25 min **Resistance component** Low–moderate load (60–70% 1-RM)^a^ Resistance training 20–25 min; 3–6 × 12 reps (3 exercises)	3 sessions/week **Aerobic component** Moderate intensity (60–70% HRR)^a^ Continuous aerobic exercise 25 min **Resistance component** Moderate load (65–70% 1-RM)^a^ Resistance training 25 min; 3–6 × 12 reps (3 exercises)	3 sessions/week **Aerobic component** Moderate–vigorous intensity (70–75% HRR)^a^ Continuous aerobic exercise 25 min **Resistance component** Moderate–high load (70–75% 1-RM)^a^ Resistance training 25 min; 3–6 × 12 reps (3 exercises)	3 sessions/week **Aerobic component** Vigorous intensity (75–80% HRR)^a^ Continuous aerobic exercise 25 min **Resistance component** High load (75–80% 1-RM)^a^ Resistance training 25 min; 3–6 × 12 reps (3 exercises)

^a^Sessions will increase in intensity/load by 2.5–5% as tolerated between sessions during weeks 1–12 up to the max intensity/load for the fortnightly block, provided the duration (during weeks 1–6) remains the same for the sessions where intensity/load is changed

^b^Sessions will increase in duration by 5 min during weeks 1–6 up to the max time for that fortnightly block, provided the intensity/load remains the same for the session where duration is increased

##### Resistance

This intervention consists of 3 × 50-min sessions per week of progressive resistance exercises. During weeks 1–6, session length will gradually progress until participants can perform 3 × 50-min sessions in week 7 (Table 3). The resistance programme contains eighteen exercises, with an equal amount of upper limb (shoulder press, bench press, bicep curl, raise, triceps exercises, bent-over row) core (seated and standing twists, hip bridge, 4-point kneel, overhead ball slam, push-ups) and lower limb (barbell squat, goblet squat, deadlift, Romanian deadlift, lunge, lateral lunge) exercises included. In each session, participants will perform six exercises aimed at targeting all major muscle groups (Table 4). The participants will perform three different exercise regimes on a weekly basis, alternating every second week (Table 5). They will be monitored to track weights lifted and be provided with feedback on correct technique as needed. In weeks 1–2, participants will perform 2 × 12 reps of the six exercises at 40–50% 1RM to allow them to become familiar with the different exercises. From weeks 3 to 4, participants will perform 3 × 12 reps of each exercise at 50–55% 1RM. From weeks 5 to 12, participants will perform up to six sets of each exercise to meet the prescribed time for each session (i.e. 50 min). The load will progressively increase for all exercises performed from weeks 1–12 from 40% 1RM in week one to 80% 1RM in week twelve (Table 3) once a participant can perform all sets of 12 reps with proper form in one session and/or once the participant reports finding the exercise easy. Each set of exercises will be followed by a 45-s rest.

**Table 4:** Resistance exercises included in the resistance and combined programmes

Exercise	Variations
**Upper body**	
Bench press^a^	Standard flat bench press with BB/DB Incline bench press with BB/DB Grip: wide, normal, narrow
Shoulder press	Overhead press with DB BB press Grip: wide, narrow Position: standing, seated
Bent over row	Standard BB row Single arm supported row with DB/KB
Raise	Forward raise Lateral raise Bent over fly Resistance: DB, weight plate
Bicep exercises	Curl with DB Curl with BB Position: standing, seated Grip: Supine, ‘hammer’, alternating supine and prone (‘Zottman’ curl)
Triceps exercises	Standing/seated behind the head extensions without resistance or 2xDB Lying triceps extensions French press with DB/KB Bench dips (BW or weight plate) KB/DB halos
Kettlebell swings	Two-hand ‘Russian’ swing Alternating swing Single hand swing Overhead ‘American’ swing
**Core**	
Push-ups	Wall push-up Elevated push-up on bench Push up on knees Push up on KB Standard push-up Position: wide hand position, narrow hand position
Seated twist	Sitting with legs supported BW Sitting with legs supported with medicine ball/ KB Sitting with legs lifted off ground and crossed BW Sitting with legs lifted off ground and crossed with medicine ball/ KB) Sitting with legs lifted off ground and held together (BW, with medicine ball/ KB)) Position: variance in torso lean back
Overhead medicine ball slam	Position: depth of squat Resistance: weight of medicine ball
Hip bridge	Two-legged bridge BW Two-legged bridge with resistance (DB, weight plate or bar) Single leg bridge with heel support of other foot Single leg bridge
4-point kneel (swimming exercise)	Slide one leg back along mat with/ without leg lift Opposite hand opposite leg (with hand still in contact with mat) Opposite hand opposite leg lift With weight plate in hand Pull-through with KB/DB/weight plate
Opposite leg touch (standing twist)	Twist to touch opposite knee up Twist to touch opposite leg kicked out Twist with weight plate in hand
**Lower body**	
Squat^a^	Chair/bench squat (BW, with KB/DB, with BB) DB/KB ‘Goblet’ squat BB Back squat BB Front Squat Split squat (one foot behind on floor; one foot supported on bench) Foot position: hip width, sumo (wide)
Lunges	Forward/reverse/side lunge Resistance: BW, Single DB/KB held in front; double DB/KB racked at shoulder or held down by sides; BB on back Position: stationary, walking lunge
Deadlift	Standard BB deadlift Sumo deadlift Romanian deadlift with DB or BB
Kettlebell swings	Two-hand ‘Russian’ swing Alternating swing Single hand swing Overhead ‘American’ swing

*BB* barbell, *BW* body weight, *DB* dumbbell, *KB* kettlebell

^a^All participants in resistance or combined programme will squat/bench or progress to squat/bench with BB unless limited physically (i.e. by injury etc.). Variations with KB/DB will be used during consecutive sessions for bench/squat

**Table 5:** Sample exercise plan

Week ‘A’		
**Session 1** Bench press with BB Bent over row Seated twist Overhead ball slam Squat with KB/DB Side lunge	**Session 2** Shoulder press Raise/KB swing Push-up Hip bridge Squat with BB Romanian deadlift	**Session 3** Biceps exercises Triceps exercises Standing twist 4-point kneel Deadlift with BB Lunge
Week ‘B’		
**Session 1** Shoulder press Biceps exercises Push-up Seated twist Squat with BB Romanian deadlift	**Session 2** Bench with BB Raise/KB swing 4-point kneel Hip bridge Squat with DB/KB Lunge	**Session 3** Bent over row Triceps exercises Standing twist Overhead ball slam Deadlift with BB Side lunge

##### Combined

This intervention will consist of 3 × 50-min sessions per week comprising of 25 min of progressive aerobic exercise (cross trainer, cycling) and 25 min (three exercises: one upper limb, one core, one lower limb) of a progressive resistance programme (Table 4). The resistance portion of this intervention can include any of the eighteen exercises outlined in the resistance intervention above. However, the prescription of compound exercises (bench press, squat etc.) will be prioritised each week over the inclusion of any single joint movements (i.e. triceps exercises). In line with the aerobic and resistance trial arms, both components (aerobic and resistance) of the combined intervention will increase in load/intensity across the 12 weeks from 40% 1RM/HRR in week 1 to 80% 1RM/HRR in week 12 (Table 3).

##### Control

The control group will be instructed to maintain their physical activity levels for the 12-week intervention period. After this period, all participants randomised to the control group will be offered the opportunity to join one of the three exercise groups as per their preference to avail of an individualised and supervised exercise programme for 12 weeks.

#### Sample size

As this is a pilot study, which has the primary goal of evaluating the feasibility of the interventions and not ensuring there is adequate power to detect between-group differences, a formal sample size calculation is not required [48]. However, the researchers recognise that the sample size will need to be large enough to provide a degree of accuracy around the estimated effect of the interventions on a desired outcome (i.e. between-group difference in CRF which would be the primary outcome in an RCT) which would indicate whether a definitive RCT is worth pursuing. We estimated that we would need to recruit approximately 10% of the number of participants needed for a full-scale RCT. Based on previous research in this area [67], a sample size calculation for a definitive RCT indicates that we would need to recruit 484 participants. Therefore, allowing for 20% attrition, we aim to recruit 60 participants for this pilot study to inform the power analysis for a full-scale RCT.

#### Blinding

Blinding of participants to their group allocation will not be possible due to the nature of the interventions. However, all participants will be informed (information leaflet) that they have an equal chance of being randomly allocated to any one of the four groups. Once the intervention commences, blinding of the assessors and instructors will not be possible. However, the assessor and exercise instructors will not be involved in group allocation and will remain unaware of the unique participant numbers until completion of the data analysis. The trial statistician will also be unaware of group allocation until completion of data analysis.

#### Statistical methods

To evaluate the feasibility of the intervention, the number of eligible participants recruited, randomised and retained as well as dropouts lost to follow-up will be analysed as percentages. Data on participants attendance adherence to and compliance with prescribed interventions (i.e. average adherence to reps, sets, HR) will be analysed and the mean results reported as percentages. The incidence of adverse events (an adverse event defined as an event which a participant identifies a problem caused by the exercise programme that required the participant to seek treatment from a health professional and/or prevents participation in the programme) will also be analysed as percentages across all interventions. The acceptability of trial procedures based on participants’ experiences will be explored qualitatively as outlined in this protocol below. Feasibility data will be used to inform the development of a potential full-scale RCT.

In line with CONSORT guidance, the analysis of secondary outcomes will be conducted on an intention to treat basis. All data collected from participants (sociodemographic information, outcome questionnaire scores etc.) will be coded, cleaned and checked for errors. The data will then be entered into the Statistical Package for the Social Sciences (SPSS Version 26) for analysis. The study’s statistician (C.B) will remain blinded to group allocation until the analysis is complete. The change in mean values before and after intervention will be calculated for secondary outcomes. Analysis of variance (ANOVA) and *t*-tests will be applied to compare between-group and within group-differences at baseline and follow-up for each outcome. If there are sufficient participants (*n* = 60), the data will be analysed using analysis of covariance (ANCOVA) with the age and BMI as covariates. Statistical significance will be defined as *p* ≤ 0.05.

#### Follow-up assessment

During the last week of the 12-week intervention period, all participants will be approached by the trial coordinator to arrange an appointment for a follow-up assessment within seven days post-intervention. Similar to the baseline assessment, participants will be instructed to attend ISH on a separate day after the intervention has ended to have their follow-up assessment following the procedures detailed above. The participants will also be issued the combined set of valid and reliable self-reported outcome questionnaires used pre-intervention.

### Qualitative study

All participants randomised to the exercise interventions will be provided an exit questionnaire and invited to participate in a semi-structured telephone interview (or face-to-face interview if preferred/convenient) within two weeks of completing their follow-up assessment. The exit questionnaire was internally developed specifically for this study and will explore participants perception of the intervention including topics such as the randomisation process, intervention design (i.e. number of sessions etc.) and satisfaction with the intervention. We aim to conduct a minimum of 18 (6 per exercise group) semi-structured interviews. Interviews will take approximately 30–40 min. Participants who consent to take part in an interview will either be contacted by phone at their convenience or invited to attend ISH for a face-to-face interview and will discuss a predetermined set of topics. These topics will be guided by the theoretical framework of acceptability (TFA) domains [68] and will include reasons for participation in the trial, their understanding and the acceptability of the trial procedures, their experiences and feelings around the exercise programmes, their expectations and satisfaction with the programmes, the barriers/motivators to participation in the trial/exercise in general, self-efficacy, changes to lifestyle, behaviours, beliefs or feelings following participation in the trial and the likelihood of continuing participation in exercise. The interview sessions will be audio-recorded and then transcribed verbatim for analysis of themes.

#### Data analysis

In line with Braun and Clarke’s method for thematic analysis [69], qualitative data collected from semi-structured interviews and exit questionnaires will be coded and analysed. Themes and sub-themes will be identified by two members of the research team. To ensure reliability of themes, an independent investigator not otherwise involved in the study will review a sub-set of the transcripts (10%) and consensus reached. Questions included in the exit questionnaire which utilises a Likert scale will be scored and the overall percentage of participants who selected specific statements will be reported.

## Results

Participant flow through the study is detailed in Fig. 1. Recruitment of participants will begin in August 2021 and is expected to last until December 2022. Data analyses are expected to begin in early 2023.

## Discussion

We have presented the rationale and design of a pilot randomised controlled trial which will investigate and evaluate the effectiveness of different training modalities (aerobic, resistance and combined) in women living with obesity compared to a non-exercise control. The results of this study will help inform exercise prescription for women living with obesity and will be presented as soon as they are available.

## Data Availability

Data of relevance to the reported findings are included in the main body of the paper with additional data provided as supplementary information. Datasets generated and/or analysed during this study will be made available in an online repository following the conclusion of the study. During the course of the study, all electronic data will be stored on the institution’s encrypted drive and on an institution’s encrypted computer. Along with the hard copies of the data, this computer will be stored in a locked filing cabinet in a locked office in the institution which only the research team have access to.

## Abbreviations

5RM: Five repetition maximum
%BF: Percentage body fat
ANOVA: Analysis of variance
ANCOVA: Analysis of covariance
BMI: Body mass index
CONSORT: Consolidated Standards of Reporting Trials
CRF: Cardiorespiratory fitness
F.I.T.T.: Frequency, intensity, time and type
FFM: Fat-free mass
FM: Fat mass
HR: Heart rate
ICPO: Irish Coalition for People Living with Obesity
ISH: Institute for Sport and Health
LM: Lean mass
PAR-Q+: Physical Activity Readiness Questionnaire for Everyone
RCT: Randomised controlled trial
RPE: Rating of perceived exertion
SPIRIT: Standard Protocol Items: Recommendations for Interventional Trials
SPSS: Statistical Package for the Social Sciences
TFA: Theoretical framework of acceptability
UCD: University College Dublin
VO_2_: Oxygen uptake
VO_2_ max: Maximal oxygen uptake
WHR: Waist-hip ratio
WC: Waist circumference
